# The relationship between e-Health literacy and cyberchondria in Iranian students of health sciences

**DOI:** 10.3389/fpsyt.2024.1421391

**Published:** 2025-02-13

**Authors:** Amirreza Kalantari, Saeideh Valizadeh-Haghi, Vladan Starcevic, Azam Shahbodaghi, Shahabedin Rahmatizadeh, Farid Zayeri, Yasser Khazaal

**Affiliations:** ^1^ Department of Medical Library and Information Science, School of Allied Medical Sciences, Shahid Beheshti University of Medical Sciences, Tehran, Iran; ^2^ University of Sydney, Faculty of Medicine and Health, Sydney Medical School, Nepean Clinical School, Sydney, NSW, Australia; ^3^ Department of Psychiatry, Nepean Hospital, Penrith, NSW, Australia; ^4^ Department of Health Information Technology and Management, School of Allied Medical Sciences, Shahid Beheshti University of Medical Sciences, Tehran, Iran; ^5^ Proteomics Research Center and Department of Biostatistics, School of Allied Medical Sciences, Shahid Beheshti University of Medical Sciences, Tehran, Iran; ^6^ Department of Psychiatry, Lausanne University Hospital and Lausanne University, Lausanne, Switzerland

**Keywords:** cyberchondria, electronic health literacy, health literacy, anxiety disorders, consumer health information

## Abstract

**Introduction:**

Cyberchondria has been growing in recent years. Understanding the relationship between e-Health Literacy and Cyberchondria is important, as enhancing e-Health Literacy perhaps empower individuals to navigate online health information without experiencing Cyberchondria. The present study aimed to investigate levels of e-Health Literacy and Cyberchondria in Iranian students of health sciences and examine the relationship between e-Health Literacy and Cyberchondria.

**Methods:**

To assess this, a sample of 241 undergraduate students of health sciences were recruited. They were administered two instruments for the assessment of e-Health Literacy and Cyberchondria: eHealth Literacy Scale (eHEALS) and Cyberchondria Severity Scale (CSS).

**Results:**

Findings showed that the mean total CSS score was 72.61, while the mean total eHEALS score was 28.50. Students who reported having a chronic disease had a significantly higher mean total CSS score than those who considered themselves healthy. The correlation between the total eHEALS scores and total CSS scores was very weak (r = -0.074). Total eHEALS scores correlated significantly and negatively with the distress (r = -0.288) and compulsion (r = -0.163) CSS subscales and significantly and positively with the reassurance (r = 0.174) and excessiveness (r = 0.141) CSS subscales.

**Conclusion:**

The relationship between e-health literacy and cyberchondria is complex. While people may develop cyberchondria irrespective of the level of their e-health literacy, a high level of e-health literacy may be protective in terms of alleviating distress and problems in functioning that occur with excessive online health searches. These findings are likely to inform future studies in this area.

## 1 Introduction

The World Wide Web is a popular resource for accessing health information (1). Its capabilities facilitate the search for medical symptoms, conditions, and treatment methods (2). In recent years, the Coronavirus (Covid-19) pandemic has led many people worldwide to search for health information online. Studies confirm that searching for medical information has become more common during this period (3, 4).

Searching for health information online has both benefits and drawbacks (5). It can empower people to use medical care (6, 7), make appropriate medical decisions (8, 9), and inform them about diseases (9). On the other hand, poor quality of online health information and ways in which people perform searches can heighten health anxiety (10, 11) and contribute to cyberchondria (12–14).

Cyberchondria is defined as an increase in anxiety or distress about one’s health following online health searches (12, 15). In other words, instead of being experienced as empowering and rewarding, online health searches become problematic (13). The negative consequences of cyberchondria include heightened anxiety and worry that may result in frequent visits to various clinicians and unnecessary investigations, which impose additional costs on the healthcare system (16). According to the most frequently used instrument for measurement of cyberchondria, the Cyberchondria Severity Scale (CSS; 17), it is assessed on five subscales. These subscales, which represent dimensions of cyberchondria, include compulsion, distress, excessiveness, reassurance, and mistrust of medical professional.

It has been suggested that prevention and management of cyberchondria should include empowering individuals to search for online health information without experiencing anxiety (13). One potential way of doing so is improving e-health literacy. E-health literacy is defined as the ability to search, find, understand, and appraise health information from electronic sources and optimally apply knowledge gained to solve health problems (17). This literacy consists of a set of various literacy skills and skills to empower people to use online health information properly (17).

Several studies have examined the relationship between cyberchondria and e-health literacy. In a study conducted among Polish internet users, it was demonstrated that high levels of e-health literacy did not protect against cyberchondria (18) The study was conducted among Polish internet users and Short Cyberchondria Scale (SCS) and e-Health Literacy Scale (eHEALS) was used for the study (18). In another study conducted among students in 8 educational centers in China using Cyberchondria Severity Scale (CSS) and eHEALS a positive and significant relationship between cyberchondria and e-health literacy was found (19). Furthermore, a study of women attending an outpatient gynecology clinic of a state hospital in Ankara, Turkey, revealed a moderate and significant correlation between cyberchondria and e-health literacy and showed that e-health literacy was a significant predictor of cyberchondria (20). Another Turkish study conducted in healthcare workers showed weak, but significant correlations between e-health literacy and excessiveness and reassurance subscales of the CSS (21).

The present study aimed to investigate the levels of e-health literacy and cyberchondria among Iranian students of health sciences, with a primary focus on examining the relationship between e-health literacy and cyberchondria within this sample. Previous research indicates that the relationship between cyberchondria and e-health literacy varies across different populations, suggesting that it can be complex. Therefore, this study seeks to conduct a detailed analysis of the relationship between cyberchondria and e-health literacy, including the specific subscales of cyberchondria, to determine whether a higher level of e-health literacy can mitigate cyberchondria or its specific disorders.

Additionally, the study focuses on health sciences students due to their regular exposure to online health information. This makes it essential to explore their e-health literacy and levels of cyberchondria, as well as the potential correlation between the two. Based on previous research findings, we hypothesized that e-health literacy would have a significant and mainly positive relationship with the construct of cyberchondria as a whole or with some of its dimensions.

## 2 Materials and methods

### 2.1 Participants and procedure

The population for this study consists of undergraduate students at Shahid Beheshti University of Medical Sciences (SBMU) in Tehran. Five SBMU-affiliated schools - Nursing and Midwifery, Public Health and Safety, Nutrition Sciences and Food Technology, Rehabilitation Sciences, and Paramedical Sciences - admit undergraduate students. At the end of the second semester of the 2020-2021 academic year, the total student population was 2718. The sample size was determined based on a pilot sample of 30 participants from the population. The correlation coefficient between the two questionnaires was calculated using this pilot sample, and the required sample size for the main study was then calculated using the following formula (PPS) with α = 0.05 and β = 0.1:


n=(Zα2+Zβ)2(12log1+r1−r)2+3

Zα2 represents the critical value from the standard normal distribution associated with the significance level α. It's used in hypothesis testing, and α2 corresponds to the two-tailed test. Zβ is the critical value from the standard normal distribution corresponding to the test's power (1−β). Power (1−β) is the probability of correctly rejecting a false null hypothesis. is the correlation coefficient, which measures the strength and direction of a linear relationship between two variables. $\frac{1}{2}log\frac{1+r}{1-r}$ is the Fisher Z-transformation of the correlation coefficient *r*. It's used to stabilize the variance of the correlation coefficient, making it more suitable for hypothesis testing. (+3) is a correction or adjustment factor added to the sample size to account for some specific conditions or to meet certain assumptions in the study design. All In all, the formula is for sample size calculation in the context of statistical hypothesis testing, specifically for correlation coefficients (22).

Based on the pilot sample, the estimated correlation coefficient was determined to be r = 0.15. Using probability proportional to size (PPS), a sample of at least 232 undergraduate students was required. The Sociodemographic Characteristics and Health Status of the sample are represented in **Table 1**.

**Table 1:** Sociodemographic characteristics and health status of the sample.

Demographic Characteristics	Groups	N (%)
Gender	Female Male	144 (60%) 97 (40%)
School	Nursing & Midwifery Public Health and Safety Paramedical Sciences Rehabilitation Sciences Nutrition Sciences & Food Technology	104 (43.2%) 40 (16.6%) 33 (13.7%) 32 (13.3%) 32 (13.3%)
Age group	18-20 20-22 22-24 <24	83 (34.4%) 112 (46.4%) 36 (15%) 10 (4.2%)
Academic Year	1 2 3 4	55 (23%) 67 (28%) 65 (27%) 54 (22%)
Self-declared Health Status	Having a chronic disease Being healthy	22 (9%) 219 (91%)

### 2.2 Data collection

Data were collected using two questionnaires: the Cyberchondria Severity Scale (CSS) and the eHealth Literacy Scale (eHEALS). In addition, sociodemographic information, including age, gender, school of study, and academic year, was collected. Participants were also asked about their health status, with the options of declaring a chronic disease or good health (self-declared health status).

Questionnaires were created in Google Docs and distributed via WhatsApp Messenger to 300 undergraduate students. This platform was chosen due to its widespread use among the student population, ensuring efficient reach and engagement. Additionally, students were also notified via their university email about the questionnaire link to ensure broader participation and mitigate potential bias from relying solely on WhatsApp. Of the students contacted, 241 (80.33%) participated in the research and provided analyzable data.

#### 2.2.1 Cyberchondria Severity Scale

The Cyberchondria Severity Scale (CSS) was used to assess cyberchondria (23). This questionnaire has been validated in Iranian population by Sarafraz et al. (24). The 33-item CSS consists of five subscales, including mistrust of medical professional, compulsion, distress, excessiveness, and reassurance. Compulsion relates to interference of online health searches with other aspects of life (both online and offline) (e.g., CSS item “Researching symptoms or perceived medical conditions online interrupts my offline work activities”). Distress refers to the perceived stress associated with online health searches (e.g., CSS item “I start to panic when I read online that a symptom I have is found in a rare/serious condition”). Excessiveness denotes multiple and repeated online searches for health information (e.g., CSS item “I read different web pages about the same perceived condition”). Reassurance relates to online health searches that drive people to seek out professional medical advice (e.g., CSS item “Researching symptoms or perceived medical conditions online leads me to consult with other medical specialists (e.g., consultants)”). Mistrust of medical professional refers to a lack of alignment between a medical professional’s advice and online health searches (e.g., a reverse-scored CSS item “I take the opinion of my GP/medical professional more seriously than my online medical research”).

Each question is set on a 5-point Likert scale (from Never to Almost Always with a score of 1 to 5, respectively) with a total score range of 33-165. Accordingly, a higher score indicates a higher level of cyberchondria. It should be noted that all 3 items of the subscale of mistrust of medical professional are reversely scored (23). The maximum scores are 40 for the compulsion, distress, and excessiveness subscales, 30 for the reassurance subscale, and 15 for the mistrust of medical professional subscale.

In addition, we calculated “relative means” for all CSS subscales as a measure of the impact of these subscales on the total CSS scores. Relative means are calculated by dividing the observed subscale means by the maximum scores on the same subscale.

#### 2.2.2 eHEALS

E-Health literacy of the study population was assessed using the eHEALS. This questionnaire assesses the ability to search, find, understand, and apply e-Health information correctly (25). The eHEALS consists of eight items set on a 5-point Likert scale (from Strongly Disagree to Strongly Agree with a score of 1 to 5, respectively) with a scoring range of 8-40. Accordingly, a higher score indicates higher e-health literacy. The eHEALS also has two supplementary items assessing the importance of accessing health information on the Internet and usefulness of the Internet in health-related decisions. The scores on these two supplementary items are not included in the total eHEALS score. The validity of eHEALS has been confirmed in Iranian population (26) and it has been used in several studies (27, 28).

### 2.3 Statement of ethics

This study protocol was reviewed and approved by the Ethics Committee of the Shahid Beheshti University of Medical Sciences (approval number: IR.SBMU.RETECH.REC.1400.260).

### 2.4 Statistical analysis

The Pearson correlation coefficient was used to examine the strength and direction of the linear relationship between e-health literacy and the overall construct of cyberchondria, as well as its individual dimensions. An independent samples t-test was conducted to compare the means of e-health literacy scores between two groups (e.g., high vs. low levels of cyberchondria) to see if there is a significant difference between them. A one-way ANOVA was used to compare the Sociodemographic Characteristics and Health Status relationships with CSS and eHEALS

Data were extracted from Google Docs in Excel file format and analyzed using the Pandas framework (version 2.0) and SciPy library in the Python programming language, version 3.10. Descriptive statistics were obtained by calculating relative and absolute frequencies for categorical variables and means and standard deviations for dimensional variables, such as total scores on the CSS and eHEALS.

## 3 Results

The mean age for the entire sample was 21.21 ± 1.32 years. Majority of participants were female (60%) and a large proportion (43.2%) were from the Nursing & Midwifery college. Most students (91%) reported being healthy, while 9% stated that they had a chronic disease at the time of their participation in the study. Relationships between cyberchondria and sociodemographic characteristics such as age, gender, school, and academic year, were not significant. Students who reported having a chronic disease had a significantly higher mean total CSS score than those who considered themselves healthy. There was no significant relationship between e-health literacy and the demographic variables. Correlations between sociodemographic characteristics and health status and CSS and eHEALS are presented in **Table 2**.

**Table 2:** Sociodemographic characteristics and health status relationships with CSS and eHEALS.

Demographic Characteristics	Groups	CSS Mean ± SD	eHEALS mean ± SD
Gender	Female Male	72.52 ± 12.80 72.74 ± 14.06	28.26 ± 4.79 28.64 ± 4.37
	F value (p)	0.122 (0.902)	0.634 (0.526)
School	Nursing & Midwifery Public Health and Safety Paramedical Sciences Rehabilitation Sciences Nutrition Sciences & Food Technology	72.04 ± 12.94 75.33 ± 12.33 71.91 ± 12.35 73.88 ± 16.49 70.56 ± 13.21	28.27 ± 4.87 28.27 ± 3.90 28.27 ± 4.94 28.53 ± 4.58 29.09 ± 4.52
	F value (p)	0.746 (0.560)	0.214 (0.930)
Age group	18-20 20-22 22-24<24	70.75 ± 12.63 74.56 ± 13.29 72.83 ± 13.98 65.50 ± 13.67	28.13 ± 4.24 28.64 ± 4.86 28.50 ± 4.07 28.00 ± 7.01
	F value (p)	2.343 (0.073)	0.222 (0.880)
Academic Year	1 2 3 4	72.36 ± 12.48 70.15 ± 14.05 74.31 ± 14.23 73.89 ± 11.78	28.38 ± 4.09 28.30 ± 5.10 27.91 ± 5.02 29.22 ± 3.98
	F value (p)	1.295 (0.276)	0.822 (0.482)
Self-declared Health Status	Having a chronic disease Being healthy	81.00 ± 13.21 71.77 ± 13.04	27.36 ± 5.63 28.53 ± 4.51
	F value (p)	3.160 (0.001)	-1.123 (0.262)

**Table 3** shows that the mean total CSS score among undergraduate students at SBMU was 72.61. The scores for each dimension are also presented in this table. The excessiveness subscale had the greatest impact on the total CSS score (0.56), followed by reassurance (0.51). The compulsion dimension had the lowest impact on the total CSS score (0.30).

**Table 3:** Students’ cyberchondria severity scale scores.

Variables (Score Range)	Minimum	Maximum	Mean ± SD	Relative mean
Cyberchondria total score (33-165)	35	119	72.61 **±** 13.29	0.44
Compulsion (8-40)	8	29	12.04 **±** 4.56	0.30
Distress (8-40)	8	33	16.26 **±** 5.35	0.40
Excessiveness (8-40)	8	40	22.75 **±** 4.69	0.56
Reassurance (6-30)	6	30	15.44 **±** 4.52	0.51
Mistrust of Medical Professional (3-15)	3	15	6.11 **±** 2.52	0.40

**Figure 1:**
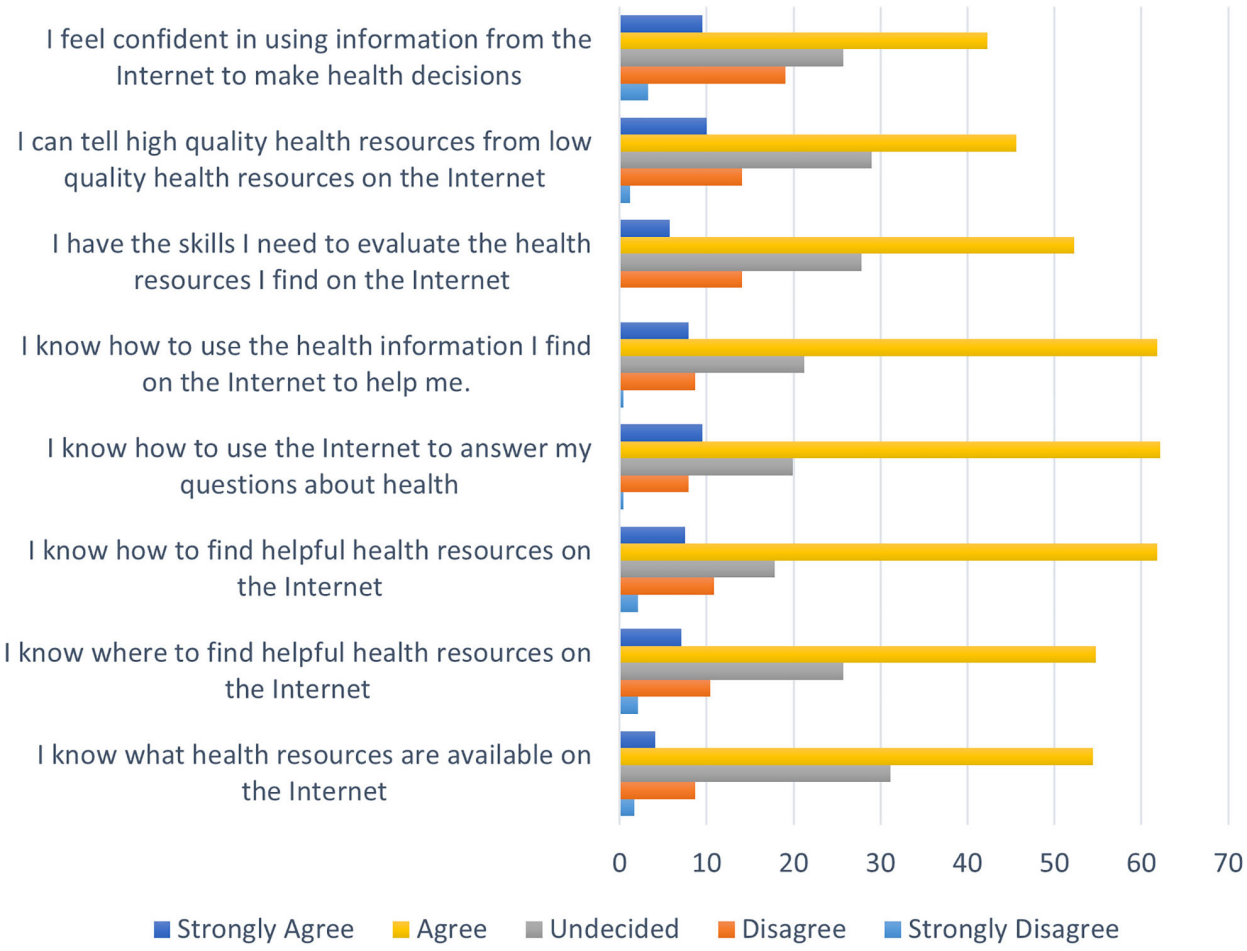
Frequency distribution of students' responses to different items of the e-HEALS.

**Table 4** shows the means and standard deviations of eHEALS items. The total mean eHEALS score in the study sample was 28.50. Given that the maximum total score on eHEALS is 40 and a score above 35 indicates an acceptable level of e-health literacy, the total mean score suggests that undergraduate students at SBMU have a low level of e-health literacy. The findings also showed that the item "I feel confident in using information from the Internet to make health decisions" received the lowest score (mean = 3.35). An examination of two supplementary questions revealed that 65.2% of participants acknowledged that the Internet was helpful and vital in their health decisions, while 74.7% stated that access to health information on the Internet was important or very important to them. **Figure 1** shows the frequency of students' responses to eight items of the eHEALS.

**Table 4:** Means and standard deviations of eHEALS items.

Item	Mean	SD
I know what health resources are available on the Internet	3.50	0.78
I know where to find helpful health resources on the Internet	3.54	0.85
I know how to find helpful health resources on the Internet	3.61	0.85
I know how to use the Internet to answer my questions about health	3.72	0.75
I know how to use the health information I find on the Internet to help me.	3.68	0.75
I have the skills I need to evaluate the health resources I find on the Internet	3.49	0.80
I can tell high quality health resources from low quality health resources on the Internet	3.48	0.89
I feel confident in using information from the Internet to make health decisions	3.35	1.00
Total	28.50	4.66

**Figure 2:**
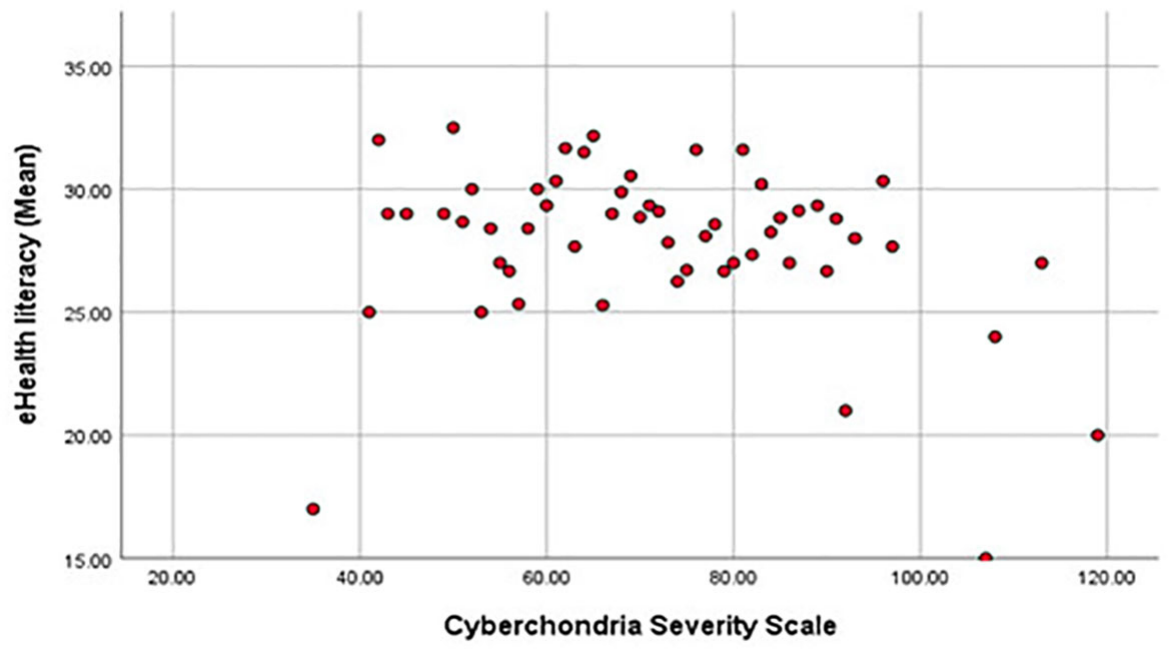
Scatter/Dot between Mean of and e-Health literacy and CSS.

**Table 5** presents the results of the Pearson Test examining the relationships between the total CSS scores, scores on its subscales and eHEALS total scores. The correlation between the total CSS score and total eHEALS score was very weak (r = - 0.074) and nonsignificant (p = 0.251). Scatter/Dot between Mean of and e-Health literacy and CSS is shown in **Figure 2**.

**Table 5:** The relationships between cyberchondria and its dimensions with e-Health literacy.

Cyberchondria subscales	Correlation coefficient	P-value
Compulsion	- 0.163	0.035
Distress	- 0.288	<0.001
Excessiveness	0.141	0.029
Reassurance	0.174	0.007
Mistrust of Medical Professional	- 0.103	0.111
Total score	- 0.074	0.251

**Figure 3:**
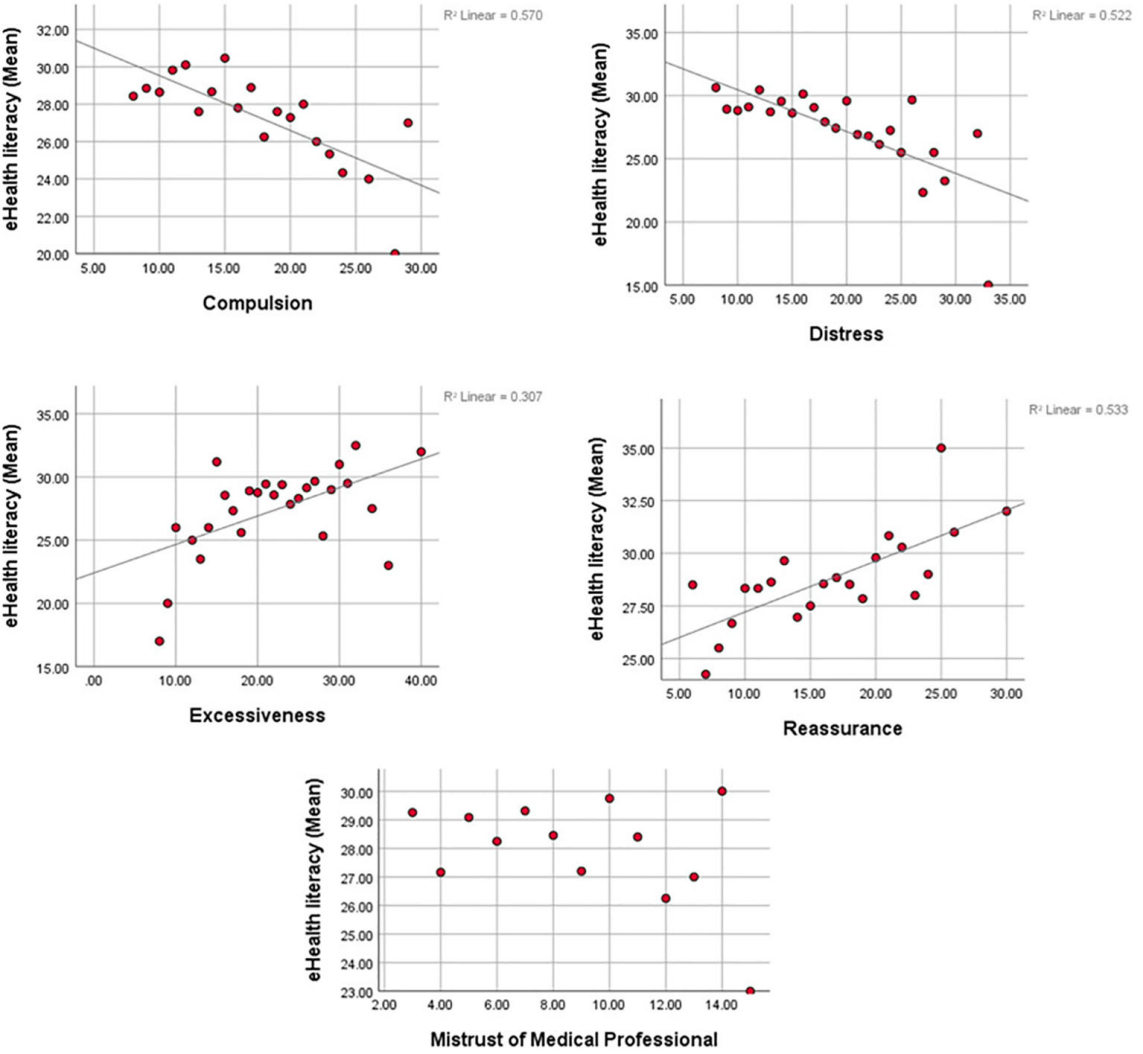
Scatter/Dot between Mean of and e-Health literacy and CSS subscales.

There were weak but significant correlations between eHEALS total score and scores on all CSS subscales, except for Mistrust of medical professional. Negative associations with eHEALS total score were observed for distress and compulsion subscales, while positive associations were observed for excessiveness and reassurance subscales. These associations are shown in **Figure 3**.

## 4 Discussion

The present study investigated levels of e-health literacy and cyberchondria in a sample of Iranian undergraduate students of health sciences and examined their correlates and associations.

Undergraduate students at SBMU exhibited a low level of e-health literacy. This was a somewhat surprising finding because students of health sciences are generally expected to have better e-health literacy. Also, students at another Iranian university demonstrated higher levels of e-health literacy (34), perhaps suggesting that there was something peculiar or unique about SBMU in terms of its ability to educate students and prepare them for searching and using online health information.

Levels of cyberchondria (mean total CSS score = 72.61) among the participants of the present study were comparable to those reported in a sample of the general population from six English-speaking countries (mean total CSS score = 72.98) (29). Other studies have also investigated levels of cyberchondria in health science or medical students (30, 31). However, it is difficult to make direct comparisons with other studies for two reasons. First, various studies used different versions of the CSS, usually with fewer items (e.g., CSS-12, CSS-15). Second, mean total CSS scores are often not published or not available.

A finding that scores on the excessiveness subscale of the CSS had the greatest impact on the total CSS scores (relative mean) is not surprising, considering that multiple and repeated online health searches are a key component of cyberchondria. Scores on the reassurance subscale also had a large impact, which can be understood in light of reassurance seeking being a common and/or important aspect of online health searches.

Participants who reported having a chronic disease had higher levels of cyberchondria than those who considered themselves healthy. This result is in agreement with the finding reported by McElroy and Shevlin (23) that individuals with higher levels of cyberchondria had more physical and mental health problems (23). The present study also reveals that demographic factors, such as age and gender, are not associated with cyberchondria, corroborating the finding by Vismara et al. (32) that consistent age and gender correlates of cyberchondria could not be demonstrated (32).

The correlation between measures of e-health literacy and cyberchondria was very weak and non-significant. This implies that individuals may suffer from cyberchondria irrespective of their e-health literacy and that greater e-health literacy does not necessarily protect against cyberchondria, when the latter is considered as a “composite” construct and assessed via total scores on the CSS. A similar finding was also reported by Rogala and Nestorowitz (19).

Important findings emerged from investigation of the relationships between e-health literacy and cyberchondria subscales. Negative correlations were found between e-health literacy and the distress and compulsion subscales of the CSS, which are the two dimensions of cyberchondria associated with its “addictive” component (29). These negative correlations were significant, but weak, suggesting that individuals with higher e-health literacy may experience less distress and interference with functioning even when engaging in online health searches excessively.

The reassurance and excessiveness subscales of the CSS exhibited positive, but weak correlations with e-health literacy, in agreement with the finding of another study (22). This may suggest that individuals with greater e-health literacy may be more prone to use online health searches for reassurance and in doing so, they may perform these searches excessively. In other words, a higher level of e-health literacy may not be sufficient to prevent excessive online health searches. The finding of a non-significant and weak correlation between e-health literacy and the mistrust of medical professional subscale of the CSS may be interpreted in light of the poor psychometric properties of this subscale due to which its items have usually been excluded from the CSS and shorter versions of the CSS in subsequent research (33–35).

The present study contributes to the body of knowledge by demonstrating that e-health literacy is related to several dimensions of cyberchondria, although it is not associated with the construct of cyberchondria as a whole. The key implication of this finding is that although a high level of e-health literacy does not necessarily protect against cyberchondria, it may alleviate distress and problems in functioning occurring with excessive online health searches. Therefore, the relationship between e-health literacy and cyberchondria seems to be complex and multifaceted. These insights are likely to inform future investigations in the area of e-health literacy and problematic online health searches.

### 4.1 Limitations

Several limitations of the present study need to be acknowledged. First, it is not certain to what extent study sample is representative of students of health sciences in Iran and students in general. Future studies should be conducted in several sites and include various groups of students. Second, reliance on self-report instruments carries the risk of reporting bias and inaccuracies in the assessment of both e-health literacy and cyberchondria. Another limitation is that the study did not take into account factors (e.g., levels of anxiety or depression) that could potentially mediate the relationship between e-health literacy and cyberchondria. Finally, the study had a cross-sectional design, which did not allow examination of any causal links between e-health literacy and cyberchondria. Future studies would therefore advance the field by adopting a longitudinal design.

## Data Availability

The raw data supporting the conclusions of this article will be made available by the authors, without undue reservation.
